# Exploring the Domains of Gender as Measured by a New Gender, Pain and Expectations Scale

**DOI:** 10.1089/whr.2020.0109

**Published:** 2021-04-09

**Authors:** Maryam Ghodrati, David M. Walton, Joy C. MacDermid

**Affiliations:** Health and Rehabilitation Sciences Program, University of Western Ontario, London, Canada.

**Keywords:** gender, gender roles, GPES, pain, psychometrics

## Abstract

***Background:*** While sex- or gender-based differences in pain expression have been documented, exploration of traditionally genderized traits on pain has been hampered by the lack of strong measurement tools. This study evaluated the structural validity of a 16-item “Gender personality traits” subscale of a recently developed Gender, Pain and Expectations Scale (GPES).

***Methods:*** Data were drawn from an existing database of 248 participants (65.7% female). Maximum likelihood-based confirmatory factor analysis was carried out while considering the conceptual meaningfulness of subscales to evaluate the factor structure identified by these traits. Construct validity was explored using *a priori* hypotheses regarding anticipated mean differences in scores between biological male and female participants.

***Results:*** A meaningful factor structure could not be defined with all 16 items. Through conceptual and statistical triangulation a three-factor structure informed by 10 items was identified that satisfied acceptable fit criteria. The factors were termed “Emotive,” “Relationship-Oriented,” and “Goal-Oriented.” Evidence of construct validity was supported through significant sex-based differences (*p* ≤ 0.02) in the expected directions for all three subscales.

***Conclusions:*** Review of the items in the three factors led the researchers to endorse a move away from naming these “masculine” and “feminine,” rather focusing on the nature of the traits: “Relationship-oriented,” “Emotive,” and “Goal-oriented.” Implications for researchers conducting sex/gender-based pain research are discussed.

Clinical Trial Registration number: NCT02711085.

## Introduction

The phenomenon of pain is multidimensional and complex, representing an almost universal experience with a high personal, societal, and global burden.^1–4^ Like any sensory and emotional experience, the experience and/or expression of pain is highly subjective and influenced by multiple interacting factors. To better understand it, exploration of the experience of pain requires consideration of psychological, biological, and cultural variables.^5–7^ There is a growing push for deep phenotyping of people in pain that will lead to more personalized treatment approaches when multiple aspects of the person are considered concurrently.^8^

One arguably under-studied phenomenon in both clinical and experimental pain research on humans is growing evidence of differences in the experience or expression of pain between men and women.^6,9–13^ Where differences have been identified, many hypotheses have been proposed, ranging from cellular mechanisms that affect nociceptive processing^14^ to culture-specific genderized social norms and roles such as stoicism and expressiveness.^14,15^ Often aligned with traditional views of masculinity or femininity, dominant cultural roles have been proposed as at least partial explanations for the findings of differences in pain expression.^6,15–21^

Sex and gender are commonly but erroneously used interchangeably in much of pain research.^22^ Sex assigned at birth does not infer gender; sex is defined as the physiological and biological characteristics of men and women such as differences in the genetic complement of chromosomes, hormones, and sex organs.^23,24^ While sex has been traditionally considered, perhaps over-simplistically, as a binary factor (male/female), gender is a more complex construct that refers to the traits and qualities of a person in relation to culturally determined norms including characteristic roles, interpersonal relationships, and expressive behaviors that can be conceptualized along a continuum.^23,25,26^ The interpretation of gender in pain research should be considered an amalgam of social norms and individual values and preferences.^23^ While gender is commonly described in terms of feminine and masculine traits, such conceptualizations seem too reductionistic. Feminist and queer scholars in particular acknowledge the non-binary nature of sex and gender^27,28^ and encourage researchers to consider both sex and gender as fluid concepts rather than rigid dichotomies.^29^ Butler (1999) and West and Zimmerman (2009) are among those who encourage a reconceptualization of gender not as a stable adjective but as a verb of “doing” and ways of behaving or becoming.^30,31^

Consideration of sex and gender in health research is increasingly acknowledged as important, evidenced partly by the Sex and Gender Equity in Research (SAGER) guidelines.^32,33^ A critical issue that has burdened research in quantitative pain science is a lack of sound measurement tools for assessing traditionally genderized traits.^24^ While different scales intended to “measure gender” have been developed such as the non-pain-specific Bem Sex Role Inventory (*BSR*I),^34^ we are aware of very few examples of its use in pain research.^35,36^ At 60 items the *BSR*I is long, and not all traits appear to be relevant to pain research.^36^ Designed four decades ago, the items on the *BSR*I are rooted in the genderized beliefs, values, and roles of 1970s America that may hold less relevance in 2020. The more recent Gender Role and Expectations of Pain (GREP) scale is intended as a more pain-oriented gender scale^37^ designed to capture self-assessment of role expectations and attributions of pain (sensitivity, endurance, and willingness to report) in relation to how respondents think of “typical” male or female behaviors. This requires respondents to have a reasonably accurate representation of how other people experience or react to pain, and also a fairly accurate understanding of how they themselves react in comparison. The psychometric properties of both scales have not been rigorously evaluated by current measurement standards and uptake in pain research appears to have been very limited to date.

In the interest of reducing the burden of capturing gender in pain research, the Gender, Pain and Expectations Scale (GPES) was developed to capture constructs included on both the *BSR*I and GREP.^38^ Using a selected subset of items from both those existing scales, the GPES is a multi-section tool wherein each section is to be interpreted separately and has its own distinct measurement properties. “Genderized personality traits” is the first section of this scale and consists of 16 items drawn from the *BSR*I by researchers experienced in sex- and gender-based pain research. These were chosen for their expected alignment with more contemporary conceptualizations of genderized traits, in particular related to expressiveness and interpersonal behaviors. As it is intended to provide summative subscale scores, there are measurement properties (*e.g*., structural validity) that must be established before it can be endorsed as a sound alternative to the existing scales.

The purpose of this study was to explore the structure (factors) definable by a list of “traditionally genderized traits” under consideration for adoption as section one of the new GPES. Results were interpreted through a lens of genderized traits and qualities as the authors understood them with particular focus on their use in pain research. Structural (factorial) validity was the priority, with concurrent known-groups validity used as a supporting evaluation.

## Materials and Methods

Secondary data collected from a sample of participants in related prior studies were collapsed into a single database for this psychometric analysis. The data were drawn from ethically approved studies: the “Systematic Merging of Biology, Mental Health and Environment” (SYMBIOME) study (clinicaltrials.gov ID number NCT02711085, Western University ethics protocol 106140) comprised of working-age adults recently involved in non-catastrophic musculoskeletal traumas (*e.g*., falls, work, sports, or related injuries), and an independent existing database from healthy university-aged adults or practicing health care clinicians. Eligible participants in all studies were at least 18 years or older, did not have cancer, serious neuromuscular or other significant systemic diseases, and could read and understand (Grade 6) English. Among other study-specific questionnaires, consistent to both databases were participants completing the GPES and providing demographics including sex-at-birth and age. Forms were completed once by each participant.

The first section of the prototype GPES under study included 16 traits that aligned with genderized roles or expectations drawn from the gendered traits literature.^38^ These included seven directly from the *BSR*I (“Independent,” “Aggressive,” “Gentle,” “Leader,” “Competitive,” “Sensitive,” “Decisive,”) and nine added by the tool designer as being aligned with both potentially genderized traits and ways of experiencing or behaving in the face of pain (“Emotional,” “Confident,” “Weak,” “Tough,” “Giving,” “Accepting,” “Determined,” “Nurturing,” “Patient”).

Respondents ranked the degree to which each of the 16 traits represents how they see themselves using a five-point magnitude-based scale: (1) “not at all like me,” (2) “very little,” (3) “somewhat,” (4) “A lot,” or (5) “Extremely like me.” Additional data tools were collected but not used for the purposes of this analysis.

### Analysis

#### Preliminary analysis: data fidelity

We first identified missing data by separating the 16 items into two broad subscales based on their theoretical assignment toward either feminine or masculine traits (8 items per scale). Those with a single missing response per scale had that response replaced with the mean of the other seven items.^39–41^ Those with two or more missing responses (≥25% missing) had that subscale, for that respondent, removed from the analysis.

Floor or ceiling effects (>30% of the sample choosing either “not at all” or “extremely”)^42^ or those items with any response option selected by <10% of respondents, were identified and flagged. Decisions on how best to address these were made on an item-by-item basis.

Descriptive analyses (frequencies, means, skewness, and kurtosis) were calculated for the demographic characteristics and GPES item response frequencies. Data were explored for normality using the Kolmogorov-Smirnov test to inform the appropriate statistical test.

### Construct validity: confirmatory factor analysis

#### First model: first-order measurement model

As the items under consideration were based on a traditionally genderized dichotomy of “masculine” and “feminine” traits, we started with a hypothesis that all 16 items would load on one of two latent constructs, 8 on each. To test this hypothesis a confirmatory factor analysis (CFA) was conducted with a base model on which the 16 items were assigned to one of the two first-order latent variables (Factor 1: “Gentle,” “Sensitive,” “Emotional,” “Weak,” “Giving,” “Accepting,” “Nurturing,” “Patient”; Factor 2: “Independent,” “Aggressive,” “Leader,” “Competitive,” “Decisive,” “Confident,” “Tough,” “Determined”). Model fit of the maximum likelihood-based CFA was evaluated using standard goodness-of-fit criteria including a nonsignificant chi-square test,^43,44^ normed chi-square (χ^2^/df) less than 3,^45^ and the comparative fit index (CFI) and Tucker-Lewis index (TLI) as incremental fit indices where values above 0.90 were desirable.^4^ Root mean square error of approximation (RMSEA) was evaluated with values less than 0.08 indicating acceptable fit.^46–48^ If the model fit was poor, communalities, residual (error), and modification indices were evaluated to identify items that were problematic (not fitting the constructs) or should have error terms correlated (indicating non-random error) and the models re-tested. This initial model was intended to provide an omnibus indicator of the degree to which all 16 traits could be adequately explained by two latent constructs and identify where shared variance may exist, but was not intended to be the final structure.

From here, an iterative process of item removal and model refinement was undertaken. This process was informed by both statistical (residuals, path coefficients, and communalities) and theoretical (consideration of alternative ways to conceptualize the items as interpersonal qualities) considerations through consensus between the co-authors (two female, one male participant with different ethnocultural backgrounds). Different factorial structures were explored that included a four-factor first-order structure (relationship-oriented, emotive, goal-oriented, and confident), a four-factor second-order structure in which the four factors from the prior model were loaded onto two second-order constructs (masculinity, femininity), and finally a three-factor first-order model in which the “confident” construct was removed owing to poor statistical and conceptual fit (on further critical interpretation, confidence did not easily fit the intention of the tool). At each step, fit indicators, residuals, and communalities/covariance were explored with the intention of identifying the model that balanced statistical and conceptual meaningfulness for the intended purpose of use in pain research with parsimony to minimize respondent burden.

#### Validation step

After settling on the most appropriate factor structure we explored construct validity of the new subscales by first creating *a priori* hypotheses based on the factors identified. As the original intent of both the GPES and the two prior subscales from which items were adapted was to explore traditionally genderized traits, self-reported sex-at-birth was used as a criterion standard against which to compare differences in subscale means. Mean scores on the new subscales were compared for differences between two levels of respondent sex-at-birth (male vs. female) using an independent T-test or *U*-test depending on the nature of the data. We hypothesized that self-identified male participants would score higher on any subscales indicating more aggressive or goal-oriented traits and female participants would score higher on those containing more expressive or relationship-oriented traits.

As the final model was unknown, the sample size was estimated for models of 2, 3, or 4 latent variables and 12 to 16 observed variables. Assuming a moderate effect size and desiring 80% power to detect it, a sample of 200 participants was considered the minimum acceptable.^49–51^ Data analysis was carried out using the IBM^®^ Statistical Package for Social Sciences (SPSS)^®^ software, version 26.0 and IBM SPSS AMOS^®^ Version 25 software (SPSS, Inc., Chicago, IL).

## Results

### Preliminary analysis: data fidelity

After removing participants with >25% missing responses (*n* = 12), the entire database consisted of 248 eligible participants (15 participants per item). Table 1 provides the sample characteristics.

**Table 1.** Characteristics of the Samples

	Healthy database (*N* = 136)	MSK trauma database (*N* = 112)	Total database
Sex (%female)	66.9%	64.3%	65.7%
Mean age (SD)	30.6 (12.9)	43.7 (14.8)	35.5 (14.8)

SD, standard deviation.

Frequencies of response options for each GPES trait are presented in Table 2. The lowest value (“not at all”) option was selected <10% of the time in 14 of the 16 items and not selected at all in 3 items. On the other extreme, the highest value (“extremely”) was selected by <10% of respondents on 7 of the 16 items. Factor analysis is impaired if not impossible when one response option has a frequency of zero, hence a decision needed to be made. Through team discussion informed by data and theory, the two lower options (“not at all” and “very little”) were combined into “very little” and the two higher options (“a lot” and “extremely”) were combined to “a lot.” So the response structure became three options for each item: 1 = “very little,” 2 = “somewhat,” and 3 = “a lot” like me. This meant that the traits of “aggressive” and “weak” showed strong floor effects, but that effect was present even before collapsing the two lower response options. The traits of “independent,” “accepting,” “giving” and “determined” showed potential ceiling effects though less severe than those with floor effects. Consistent with the purpose of the tool and the researchers' positions on the value of such items, aggressive and weak were flagged for removal as, upon reflection and data distribution, both were deemed to be burdened by negative cultural stigma. All remaining items showed no strong evidence of skewness (non-significant Kolmogorov-Smirnov test) after collapsing response options.

**Table 2.** Descriptive Statistics for the Items in the Gender, Pain, and Expectations Scale

Items	Response options percent					Mean	Skewness	Kurtosis	Floor/ceiling effect
	Not at all	Very little	Somewhat	A lot	Extremely				
Independent	0.0	0.8	11.3	46.6	41.3	4.3	−0.6	−0.2	✖
Emotional	2.4	19.0	42.9	26.7	8.9	3.2	0.1	−0.3	
Aggressive	17.1	50.4	26.8	4.5	1.2	2.2	0.6	0.6	✖
Gentle	1.2	8.5	43.7	39.7	6.9	3.4	−0.2	0.2	
Confident	1.6	6.5	35.6	47.8	8.5	3.5	−0.5	0.6	
Weak	27.4	50.0	19.4	2.8	0.4	2.0	0.6	0.3	✖
Tough	2.0	13.4	43.5	32.5	8.5	3.3	−0.1	−0.1	
Leader	1.2	10.9	33.9	41.5	12.5	3.5	−0.3	−0.2	
Competitive	3.6	14.2	23.9	36.4	21.9	3.6	−0.5	−0.5	
Nurturing	0.4	9.7	32.3	37.9	19.8	3.6	−0.2	−0.6	
Accepting	0.0	4.0	26.6	48.8	20.6	3.8	−0.2	−0.4	✖
Giving	0.0	2.0	25.4	54.0	18.5	3.9	−0.2	−0.3	✖
Determined	0.4	1.2	15.3	52.4	30.6	4.1	−0.6	0.7	✖
Sensitive	1.2	9.8	35.8	39.4	13.8	3.6	−0.2	−0.2	
Decisive	5.3	19.4	40.5	29.1	5.7	3.1	−0.2	−0.3	
Patient	4.4	16.1	35.9	33.1	10.5	3.3	−0.2	−0.3	

### Construct validity: CFA

**FIG. 1.**
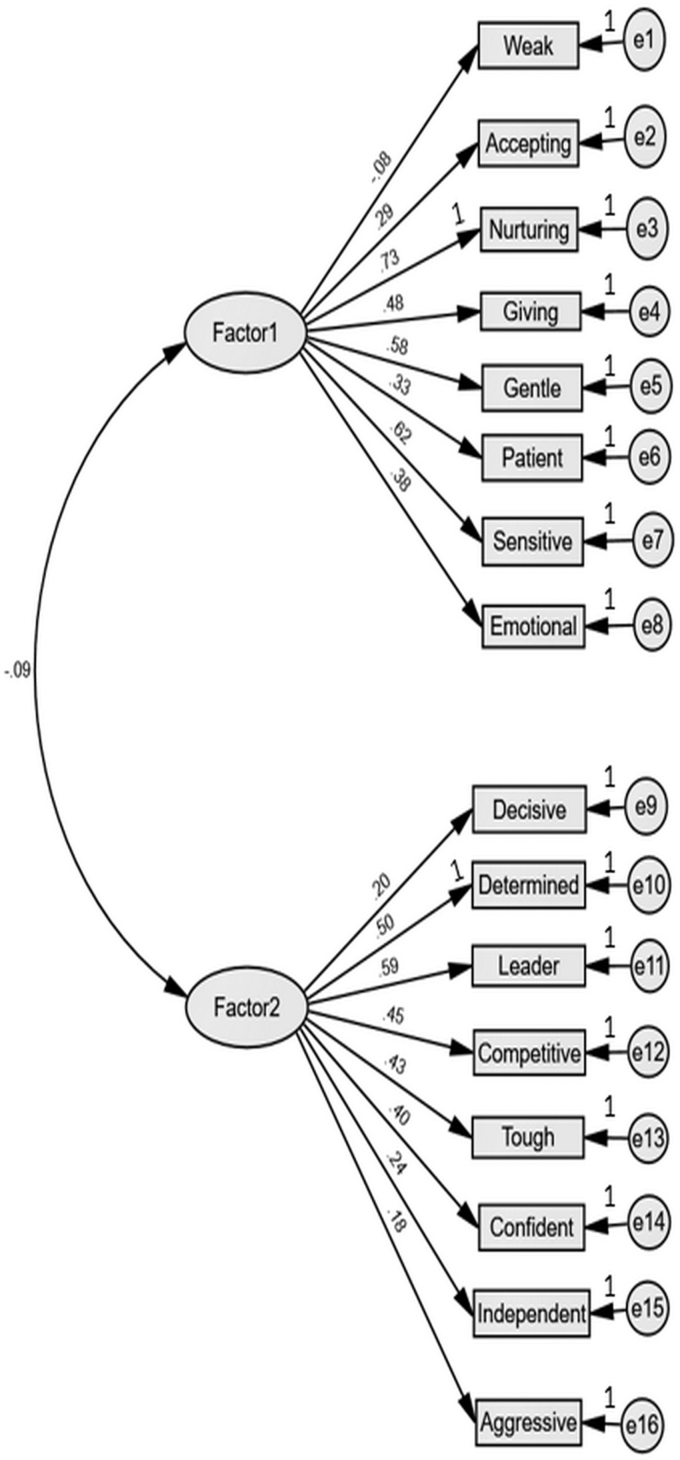
Path diagram for the CFA of the Model 1 (two first-order factors). Values are standardized path coefficients for items. The loading for each item is shown above the arrow. e = error. CFA, confirmatory factor analysis.

The path diagram for the base model is shown in Figure 1. As expected, fit indicators suggested poor model fit to the data and therefore the model was rejected (χ^2^/df = 343.560/103; CFI = 0.57; TLI = 0.50; RMSEA = 0.09).

Each of “aggressive,” “weak,” “independent,” “accepting,” and “patient” showed either very large residuals, non-significant path coefficients with their assigned construct, or strong floor/ceiling effects. These were removed and the model retested, resulting in moderate fit improvement but still under acceptable thresholds.

**FIG. 2.**
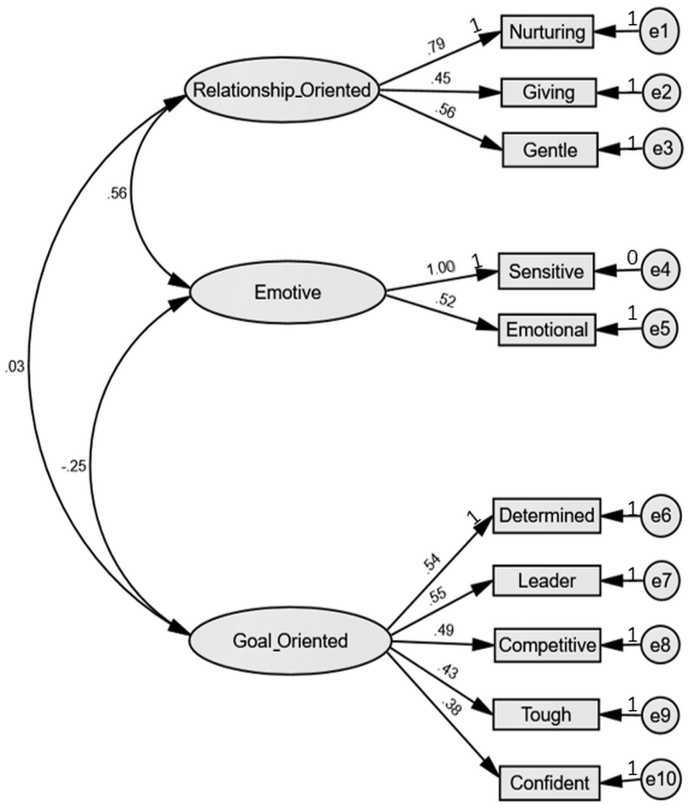
Path diagram for the CFA of the Model 4 (three first-order factors). Values are standardized path coefficients for items. The loading for each item is shown above the arrow. e = error.

The four-factor first-order model showed an acceptable fit to the data, but the four-factor second-order model did not (Table 3). In both models, the trait “decisive” showed consistently high residuals and very low path coefficients (standardized β < 0.30) regardless of the factor to which it was assigned. Again turning to theory, while “decisive” and “confident” appeared to both be informed by a latent construct of confidence in decisions, we could not achieve acceptable model fit while loading those two items onto a single construct. The “confident” item also loaded strongly onto one of the other latent constructs, hence through deliberation we removed “decisive” and loaded the remaining 10 items onto three latent constructs that were subsequently termed “relationship-oriented” (nurturing, giving, and gentle), “emotive” (sensitive, emotional), and “goal-oriented” (determined, leader, competitive, tough, and confident). This model (shown in Fig. 2) revealed good fit to the data (χ^2^/df = 1.77; CFI = 0.93; TLI = 0.90; RMSEA = 0.05, Table 3). Variance in the “sensitive” item was fixed to zero to achieve adequate convergence with no substantive effect on any other path coefficient. The Emotive factor had a positive significant correlation with the Relationship Oriented factor (*p* < 0.01), and a negative significant correlation with the Goal-oriented factor (*p* < 0.01), supporting concurrent validity. The relationship-oriented factor had no significant correlation with the goal-oriented factor. This 10-item, three-factor structure was deemed optimal in both statistical and theoretical terms and carried forward to the subsequent validation step.

**Table 3.** Summary of Fit Indices for Models of the Gender, Pain, and Expectations Scale Derived from Confirmatory Factor Analysis

Model	χ^2^ (*p*)	df	χ^2^/df	CFI	TLI	RMSEA
Model 1 (two first-order factors)	343.56 (0.00)	103	3.33	0.57	0.50	0.09
Model 2 (four first-order factors)	63.08 (0.00)	38	1.66	0.93	0.90	0.05
Model 3 (four first-order factors, two second order factors)	123.79 (0.00)	43	2.87	0.78	0.71	0.08
Model 4 (three first-order factors)	56.85 (0.00)	32	1.77	0.93	0.90	0.05

CFI, comparative fit index; RMSEA, root mean square error of approximation; TLI, Tucker-Lewis index.

### Validation step

**FIG. 3.**
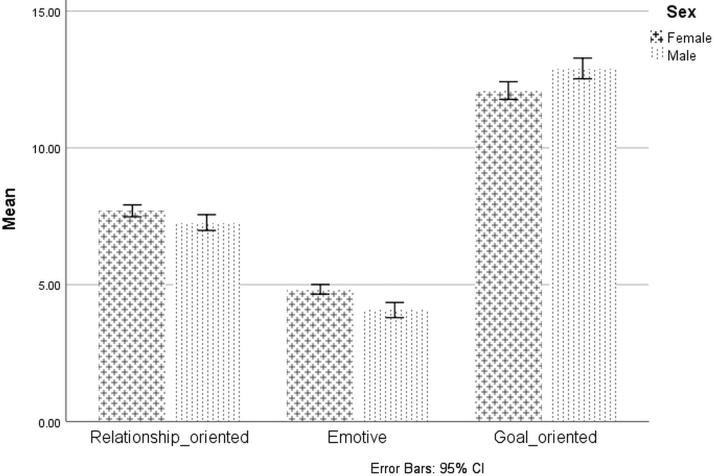
Compare mean scores of subscales (relationship-oriented, emotive, goal-oriented) in male and female. Sex-based differences are significant (*p* ≤ 0.02) for all three subscales. Error bars: 95% CI. CI, confidence interval.

The sample comprised of 85 self-identified biological male and 163 biological female participants. No participants reported a non-binary sexual identity. The data were adequately normal and of equal variance per Levene's test in both groups. An independent sample t-test revealed that mean scores on all subscales were statistically significant in the direction supported by theory. Results revealed that male participants scored significantly higher on the goal-oriented subscale [*t*(246) = −3.05, *p* < 0.001] while female participants scored significantly higher on the emotive subscale [*t*(246) = 4.74, *p* < 0.001] and the relationship-oriented subscale [*t*(246) = 2.31, *p* = 0.02] (Table 4). The clustered bar charts are presented in Figure 3.

**Table 4.** Independent Sample *t*-Test for Model 4

Sex	*N*	Mean	SD	*t*(df)	*p*
Model 4					
Relationship-oriented subscale					
Female	163	7.69	1.41	2.31 (246)	0.02
Male	85	7.27	1.33		
Emotive subscale					
Female	163	4.82	1.14	4.74 (246)	<0.001
Male	85	4.07	1.28		
Goal-oriented subscale					
Female	163	12.09	2.08	−3.05 (246)	<0.001
Male	85	12.90	1.74		

## Discussion

While the volume of sex-based research has increased, driven notably by recent advances in lab-based animal research, the socially constructed phenomena of gender roles or identities remain under-explored in pain research. While many authors have reported differences in pain expression between the sexes, other authors have reported conflicting evidence for a between-sex difference in pain sensitivity, pain endurance or threshold.^20,52–58^ It is therefore unclear what mechanisms may be driving between-sex differences, what proportion of those differences are related more to gender than sex, and how, if at all, such findings should influence clinical decisions.

This study describes a rigorous analysis of 16 traits that have been traditionally associated with gender role expectations for use in a new GPES intended to facilitate research in this area. While all 16 traits may be of value, our analyses have revealed three subscales informed by 10 of those traits with factor structures that are in keeping with our theoretical understanding. We intentionally termed the two subscales Traditionally Feminine or Masculine as we align with other gender scholars who endorse a position that these constructs are not dichotomous, and may be unintentionally harmful. Indeed, other authors have found that traditionally “masculine” and “feminine” traits might be quite similar between men and women.^59^ This may be due to a lack of sound assessment tools for gender traits, or it may be the result of a shift in how gender has been conceived and defined over recent decades.^60^ The shifting roles of women in North American society is arguably most noticeable, with epidemiological data showing clear upward trends in the numbers of women who work outside of the home, hold leadership positions, and delay childbearing until later in life.^61–63^ Accordingly, some scholars have endorsed alternative nomenclature to unlink sex from gender, toward traits such as “Interpersonal Sensitivity,” “Communion,” “Expressiveness,” or “Instrumentality” rather than Feminine or Masculine.^64–68^

The three subscales appear to resonate with the arguments of those more contemporary scholars, and hold theoretical value for exploring pain experience or expressiveness. We have intentionally used the phrase “experience or expressiveness,” as it is widely recognized that, as outside observers, it is impossible to separate the experience of the person in pain from their expressions of that pain.^69^ As such, the evaluation of another person's pain is necessarily dependent on their willingness to express (or their emotiveness), their sense of connection (or relationship) with the observer, and their drive to overcome the experience of pain to achieve certain goals. The subscales of “emotiveness,” “relationship-oriented,” and “goal-oriented” should therefore hold meaning for those conducting sex- and gender-based pain research.

The “Goal-oriented” subscale includes tough, competitive, leader, confident, and determined, traits that are likely associated with goal-oriented people that could be used to motivate others to action regardless of biological sex. “Emotive” includes sensitive and emotional, and “Relationship-oriented” includes nurturing, giving, and gentle, traits that are expected to be associated more with prosaically bonding with and supporting others. While this approach is supported by our independent samples t-tests that revealed statistically significant sex differences on the subscales, the absolute mean differences between sexes were small. Costa et al.^70^ found that women were perceived as more nurturing than men and men more assertive, supporting this as a reasonable approach to “known-groups” construct validity, but is admittedly an elementary one. By moving beyond conceptualizations of traits as either rigidly “masculine” or “feminine,” this framework allows a person to be goal-oriented, emotive and relationship-oriented all at the same time, and not inherently defined by sex. While we believe this approach to be more rooted in current cultural values, it will likely also add complexity to sex- and gender-based pain research, though also add rigor as traditional dichotomies may be overly reductionistic.

Many existing gender-based scales, whether for pain research or otherwise, have been adapted from Bem's study in 1974.^34^ These include the Personal Attributes Questionnaire (PAQ),^71^ Appropriate Pain Behavior Questionnaire (APBQ),^72^ GREP,^37^ Traditional Masculinity-Femininity Scale (TMF),^60^ masculinity and femininity Adjective Checklist scales (ACL),^73^ and Personality Research Form (PRF) ANDRO scale.^74^ For example, Myers et al.^36^ used the *BSR*I in an investigation of the influence of gender in interpreting the correlation of experimental pain and sex, and showed an association between noxious cold responses and masculinity-femininity but they opined that future work needed to build better explanations regarding sex- and gender-based pain perception, which would be very difficult with the original *BSR*I. Nayak et al. developed two distinct versions of the APBQ to find that, in U.S. and Indian cultures, expressing pain was considered more acceptable for women than for men.^72^ Pool and colleagues used the Gender Group Identification Questionnaire and Gender Norms for Pain Tolerance Questionnaire to explore differences in pain tolerance between men and women and how those were explained by self-perceptions of gender.^75^ They found higher pain tolerance in high-identifying men than high-identifying women and low-identifying men; they highlighted the role of social norms in pain reporting.^75^ As a more pain-centric tool, Robinson and colleagues developed and tested the GREP scale. The GREP has been used in a number of studies in different cultures, and commonly reveals a relationship with experimental pain across multiple stimulus modalities^6,16–18,76^ though evidence of its use in clinical pain samples is more difficult to find. We hope that the GPES, as a tool that combines self-perceived traits with beliefs and expectations of pain sensitivity, will offer a brief yet robust approach to capturing these important person- and social-level constructs.

Our findings revealed that several of the items, themselves a carefully chosen subset of the original *BSR*I traits, could not be reliably or confidently assigned to any single meaningful factor. Interestingly it seems the field has seen this several times prior using the *BSR*I; a summary of findings from 23 factor analysis studies showed that the structure of the masculine and feminine subscales was not adequate to capture the complexity of these constructs.^77^ Rose and colleagues suggested that the inherent meaning of the *BSR*I items is questionable, and may be better defined as instrumentality and expressiveness rather than the masculinity and femininity.^78^ Frable,^79^ and Holt and Ellis^80^ have both opined that the *BSR*I has been used widely without considering the appropriation of its conceptual framework for the hypotheses being studied. Others have suggested that masculinity and femininity are not simple constructs that can be conceptualized as ends of a single continuum but potentially interact in complex ways and are better suited to dedicated subscales.^81–83^ The GPES seems to be a tool that could allow such complex interactions to be explored in new ways. This position will be strengthened as we explore associations between the subscales identified herein and clinical indicators of pain severity or interference. These are planned analyses for subsequent study.

Limitations of the study include a smaller representation of male than female participants in our combined databases, and an inability to retest the revised scoring of the tool in an independent cohort. The homogeneous sampling design was not adopted in this study. While perhaps adding some statistical noise, a heterogeneous sample allows capture across a wide range of perspectives, and the arrival at a theoretically- and statistically acceptable model despite the noise suggests our fit indicators are likely more conservative than would have been seen in a more homogenous sample. The over-representation of female participants is consistent with what is known both about the population of people reporting pain after trauma, and the composition of rehabilitation training programs at our university from which the second (non-clinical) cohort was largely drawn. Testing of the revised scoring of the tool in a more diverse cultural groups is a logical next step.

## Conclusion

In light of the results of our study, the new GPES appears to be a promising general measure of personal traits that may be related to traditionally genderized factors. While the factor structure was supported with only 10 of the 16 items, we are not yet endorsing the removal of all other items because structural validity should not be conflated with importance, which has yet to be fully explored. If the reliability and validity of the GPES hold up in future studies it could be used as part of a comprehensive exploration of sex- and gender-based differences in clinical and experimental pain research in humans. However, through discussion, we have also highlighted that the traditional conceptualization of common traits as “masculine” and “feminine” is likely overly simplistic and that researchers are encouraged to consider what the important domains of these constructs are that may influence things like pain experience, reporting, or endurance.

## Abbreviations Used

ACL: adjective checklist
APBQ: Appropriate Pain Behavior Questionnaire
*BSR*I: Bem Sex Role Inventory
CFA: confirmatory factor analysis
CFI: comparative fit index
GPES: Gender, Pain, and Expectations Scale
GREP: Gender Role and Expectations of Pain
PAQ: Personal Attributes Questionnaire
PRF: Personality Research Form
RMSEA: root mean square error of approximation
SD: standard deviation
SPSS: Statistical Package for Social Sciences
SYMBIOME: Systematic Merging of Biology, Mental Health, and Environment
TLI: Tucker-Lewis index
TMF: Traditional Masculinity-Femininity
